# Two-photon microscopy allows for rapid imaging and diagnosis of Mohs specimens

**DOI:** 10.1016/j.jdcr.2025.10.042

**Published:** 2025-10-30

**Authors:** Connor M. Heckman, Chi Z. Huang, Vincent D. Ching-Roa, Sherrif F. Ibrahim, Michael G. Giacomelli

**Affiliations:** a Department of Biomedical Engineering, University of Rochester, Rochester, New York; b Department of Dermatology, University of Rochester Medical Center, Rochester, New York; c Rochester Dermatologic Surgery, PC, Victor, New York

**Keywords:** Mohs micrographic surgery, basal cell carcinoma, squamous cell carcinoma, slide-free histology, fluorescence microscopy

## Introduction

Mohs micrographic surgery (MMS) minimizes destruction of healthy tissue and achieves high tumor clearance rates through comprehensive evaluation of the surgical margin. Despite this, frozen section analysis (FSA) used for intraoperative histology in MMS is not without flaws. First, FSA involves loss of diagnostic tissue from the true margin, leading to reduced diagnostic specificity.^1^ Second, the lack of real-time feedback between tissue mounting and slide quality increases the chances for incomplete margin coverage. This, in turn, can result in repeated sectioning and slide processing, ultimately bottlenecking clinical workflow. For larger specimens, Mohs layers must first be sectioned into pieces that can fit on a standard microscope slide, each requiring separate preparation by the histotechnician. In a typical Mohs practice, it is not uncommon for a large, single excised tumor to require 4 to 12 sections to allow for full microscopic margin analysis. Also, these bigger tumors commonly contain thicker portions of fat, which further prolong freezing and preparation times. As a result, processing of large specimens consumes a disproportionate amount of clinic resources, causing long delays for the affected patient and potentially for other patients being treated on the same day.

To address these concerns, we evaluated the use of two-photon fluorescent microscopy (TPFM), a slide-free histology technique that can rapidly image Mohs specimens to produce virtual hematoxylin and eosin-stained histology images, completely bypassing the need for established gross and histologic processing techniques. Compared to other techniques, such as confocal microscopy, TPFM has the ability to image deeper into tissue due to its longer excitation wavelength at similar resolutions and speeds.^2^ In a prior study, we introduced the use of this technology to image features of both basal and squamous cell carcinoma with high accuracy.^3^ To explore the current application, diagnostic images containing subcellular resolution were obtained post-FSA from representative MMS cases, including cases of basal cell carcinoma (BCC), squamous cell carcinoma (SCC), and a large MMS case, which was processed in a fraction of the time required for conventional cryosectioning.

## Case 1

**Fig 1:**
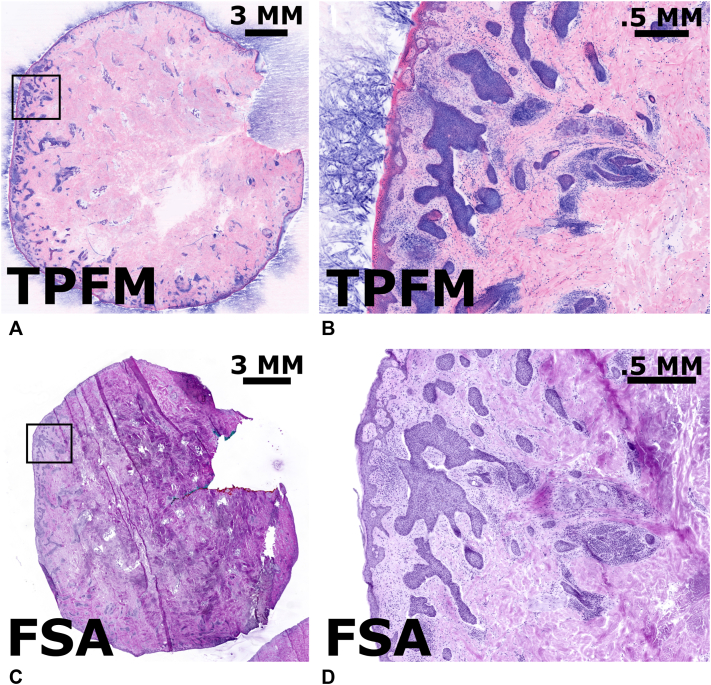
A Mohs specimen, stained with AO/SGR (nuclei) and SR101 (protein), was imaged with TPFM and found to be positive for BCC **(A)**, accompanied by a zoomed image of one region positive for BCC **(B)**. Additionally, we present the corresponding FSA section **(C)** with the corresponding zoomed region, also positive for BCC **(D)**. These images highlight TPFM’s capability to represent BCC in a comparable way to FSA. *AO*, Acridine orange; *BCC,* basal cell carcinoma; *FSA*, frozen section analysis; *SGR*, SYBR Green; *SR101*, sulforhodamine 101; *TPFM,* two-photon fluorescent microscopy.

A biopsy-proven BCC referred for MMS, measuring approximately 17.4 mm by 16 mm. Following standard Mohs histologic techniques, the specimen thawed in room temperature water and stained using 100 μg/ml sulforhodamine 101, 50 μg/ml SYBR Green, and 150 μg/ml acridine orange in phosphate-buffered saline with 20% ethanol for 3 minutes and washed in PBS for 10 seconds to remove excess stain, blood, and free-floating fat. Then, the specimen was mounted and imaged using our clinical TPFM system.^4^ During review by a Mohs surgeon, both the TPFM and FSA images were found to contain residual BCC (Fig 1). Images from the FSA and TPFM revealed an excellent degree of coregistration.

## Case 2

**Fig 2:**
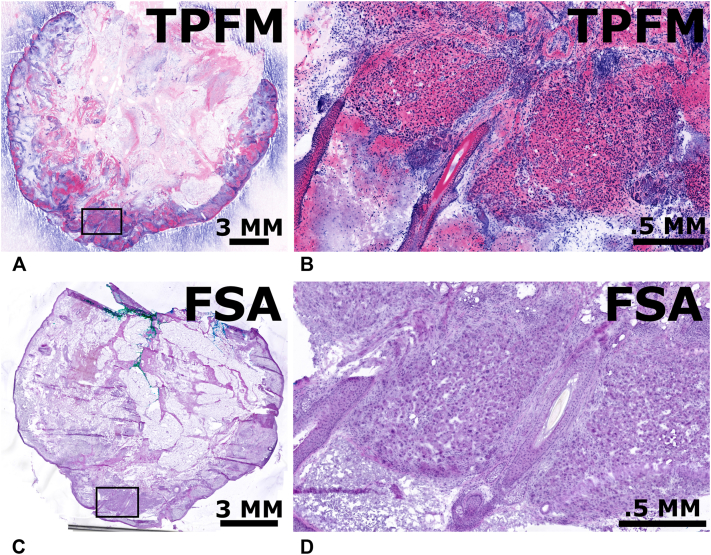
A Mohs specimen, stained with AO/SGR (nuclei) and SR101 (protein), was imaged with TPFM and found to have residual invasive SCC **(A)**, accompanied a zoomed image of one region positive for SCC **(B)**. Additionally, we present the corresponding FSA section **(C)** with the corresponding zoomed region, also positive for SCC **(D)**. These images highlight TPFM’s capability to represent SCC in a comparable way to FSA. *AO*, Acridine orange; *FSA*, frozen section analysis; *SCC,* squamous cell carcinoma; *SGR*, SYBR Green; *SR101*, sulforhodamine 101; *TPFM,* two-photon fluorescent microscopy.

A Mohs stage from a biopsy-proven SCC referred for surgery, measuring approximately 24.6 mm by 22.3 mm, was stained and imaged using the same protocol in case 1. When reviewed by the Mohs surgeon, both the TPFM and FSA showed regions containing residual SCC (Fig 2). Again, both the FSA and TPFM revealed similar features with excellent coregistration.

## Case 3

**Fig 3:**
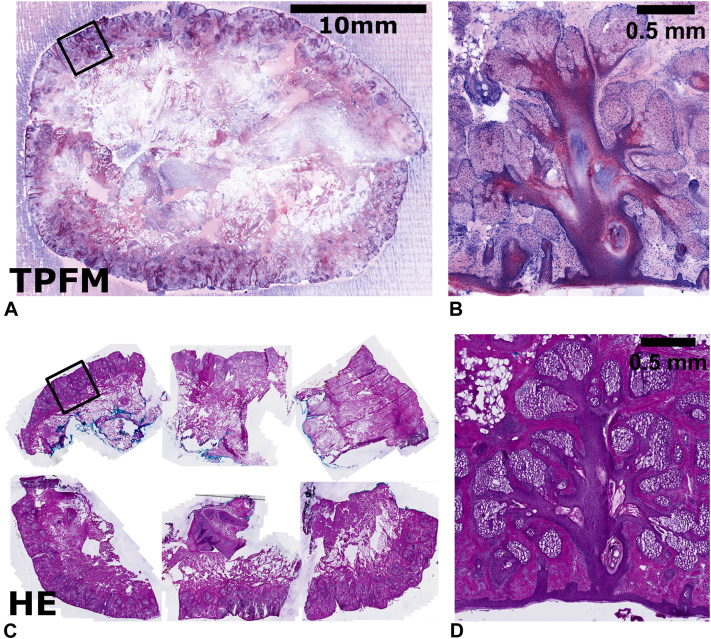
A two-photon image of a 23 × 31 mm BCC Mohs micrographic surgery sample stained with AO/SGR (nuclei) and SR101 (protein) **(A)** and a zoomed region **(B)** of a hair follicle. The sample took approximately 5 minutes to stain and mount, while imaging took 3 minutes to acquire. The corresponding H&E-stained FSA sections for the sample are shown below **(C)** with a zoomed region **(D)**. These zoomed regions depict the capability of TPFM to achieve subcellular resolution comparable to FSA. *AO*, Acridine orange; *BCC,* basal cell carcinoma; *FSA*, frozen section analysis; *H&E*, hematoxylin and eosin; *SGR*, SYBR Green; *SR101*, sulforhodamine 101; *TPFM,* two-photon fluorescent microscopy.

To demonstrate the ability of TPFM to image larger Mohs specimens, a Mohs layer measuring approximately 25 × 30 mm was divided into 6 sections and processed following standard Mohs histologic techniques. The 6 pieces were then thawed and removed from the optimal cutting temperature compound in room temperature water. Then, all pieces were stained following the steps outlined in case 1. Next, using the Mohs map, the specimens were assembled into the correct orientation to recreate the full, unsectioned Mohs layer. The specimens were then mounted and imaged with the TPFM system. Preparation time from obtaining the specimen to image generation was approximately 10 minutes and did not require any standard histologic processing. The corresponding Mohs sectioning required more than 1 hour to process, and no other specimens in the laboratory could be attended to during this time. Finally, the image was analyzed by a Mohs surgeon (Fig 3). Results showed both the TPFM images and the FSA slides were negative and contained near-perfect coregistration between the FSA slides and our TPFM images.

## Discussion

We present here 3 cases of rapid and accurate tissue processing and imaging of a Mohs layer. In 2 cases, we present the ability of TPFM to capture images of BCC and SCC pathology comparable to FSA. Although the images shown were acquired from specimens post-FSA, this protocol and imaging would not be altered for fresh specimens immediately removed from the patient and without prior sectioning. This is notable because previous studies have shown that FSA can remove 189 to 325 μm of tissue in the form of partial sections during the facing step of slide preparation.^1^^,^^5^ In contrast, TPFM imaging requires no initial tissue removal of the true deep margin of removed skin, reducing the likelihood of obtaining a positive margin and the need for additional Mohs layers.

Additionally, when using TPFM, the large Mohs specimen from case 3 was prepared in a fraction of the time required for conventional Mohs histology. This is because TPFM can allow for the processing and imaging of specimens without the need to section Mohs layers into multiple pieces, dramatically reducing the time required for specimen preparation independently of excised tissue dimensions. Currently, the maximum imaging size, 75 mm by 110 mm, is limited by the microscope’s motorized XY stage. Future designs could greatly increase this if required.

Furthermore, processing factors associated with tissue sectioning into multiple pieces, such as mislabeling of specimens, misorientation of pieces, floaters, and misaligned imaging planes, are avoided. Future applications of this procedure are not limited to large or single Mohs specimens. These include real-time biopsy evaluation, real-time assessment of standard excision margins, and other applications requiring intraoperative margin evaluation. Imaging specimens containing bone or hard cartilage, not possible with FSA, can be conducted with TPFM. It is also possible to stain and image more than one Mohs specimen in parallel, which enables further improvements to clinical workflow while providing comparable diagnostic resolution to FSA.

Finally, although the cost of fluorescent stains used in this study are relatively inexpensive, the TPFM system does require expensive optical components. Once produced in mass, we anticipate the cost approaching that of a high-end cryotome. Furthermore, these costs would be offset by no longer requiring a Mohs laboratory and the equipment, personnel, and reagents it requires as well as the reduced processing time, enabling more procedures to be performed.

## Conclusion

These 3 cases demonstrate that TPFM can provide equivalent and potentially more accurate diagnostic information while also reducing the time it takes to analyze large Mohs specimens compared to FSA. This will facilitate enhanced workflow in the Mohs clinic to prevent the common bottlenecks encountered with these specimens.

## Conflicts of interest

None disclosed.
